# The deltoid muscle and the pattern of paresis in ALS

**DOI:** 10.1007/s00415-025-12949-w

**Published:** 2025-03-06

**Authors:** Albert Ludolph, Veronika Klose, Jens Dreyhaupt, Kelly Del Tredici, Heiko Braak

**Affiliations:** 1 Department of Neurology, Ulm University, Oberer Eselsberg 45, 89081 Ulm, Germany; 2 German Center of Neurodegenerative Diseases, Ulm Site, Ulm, Germany; 3 Institute of Epidemiology and Medical Biometry, Ulm University, Helmholtzstraße 22, 89081 Ulm, Germany; 4 Clinical Neuroanatomy, Department of Neurology, Center for Biomedical Research, Ulm University, Helmholtzstraße 8/1, 89081 Ulm, Germany

**Keywords:** Amyotrophic lateral sclerosis, Biceps, Corticomotorneuronal, Deltoid, Monosynpatic, Pattern of paresis

## Abstract

There is neuroanatomical and clinical evidence that the corticospinal tract governs the patterns of pareses in sporadic ALS. These patterns are mirrored by phylogenetically young monosynaptic corticomotor neuronal connections. It is well known that, clinically, dysfunction of the deltoid muscle contributes considerably to the early disability of the ALS patient. In this study, we prospectively compared the degree of pareses of the deltoid muscle with the triceps and biceps brachii in *N* = 71 patients (426 muscles). We could show that the extent of involvement of the deltoid muscle early in the disease process resembles that of the biceps rather than the triceps brachii. This pattern is consistent with functional data of the corticospinal monosynaptic connectivity of all three muscles.

## Introduction

It has long been suspected [8, 22, 32] but also supported by human and non-human primate studies that the patterns of pareses in amyotrophic lateral sclerosis (ALS) reflect the distribution and strength of monosynaptic corticobulbar and corticospinal connections (corticomotoneuronal fibers) [1, 2, 4, 6, 11, 15, 18, 24, 28]. This is clinically true for the small hand muscles [10, 32], the muscles responsible for more proximal arm movements [14, 16, 17], but also for other muscle groups, including the leg muscles [9, 19]. Since, in particular, the deltoid muscle is likely affected early in the ALS clinical disease process [10], we tried to systematically probe this observation in a prospective study and to compare the degree of pareses with two neighboring muscles—one that is densely supplied with direct monosynaptic connections (biceps brachii) and the other (triceps brachii) less densely supplied with corticomotorneuronal fibers [14, 20]. Here, we show that the degree of paresis of the deltoid muscle closely resembles the biceps more than the triceps brachii.

## Patients and methods

### Patients

*N* = 71 patients were examined in the outpatient clinic of the Department of Neurology at the University of Ulm and diagnosed prospectively according to the El Escorial Criteria [3] by a board-certified neurologist (ACL) between April and November 2023. Each patient was thoroughly examined using a routine documentation system that included muscle strength of specific muscle groups according to the British Medical Research Council (BMRC) scale [12, 21], as described previously [20]. In the present study, deltoid muscle strength was added to the existing routine examination. The study was performed in compliance with the ethical principles originating in the latest version of the Declaration of Helsinki and approved by the local institutional ethics board (references 11/10 and 19/12). Informed written consent was obtained from all participating patients.

### Pattern of muscle weakness (paresis)

**Fig. 1:**
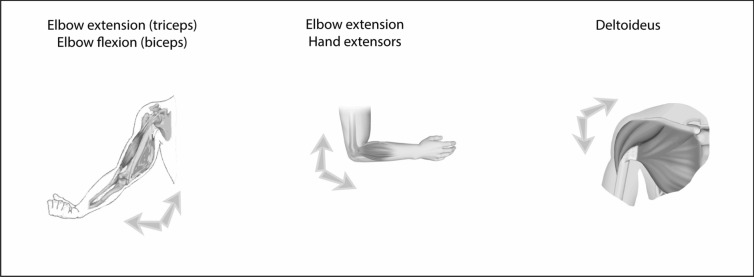
Muscle groups tested. The strength of the triceps brachii, biceps brachii, and lateral part of the deltoid muscle were compared

In all patients, muscle strength was compared following physical examination and testing of the deltoid, biceps brachii, and triceps brachii muscles (Fig. 1). The strength of the deltoid was tested by asking the patient to perform arm abduction along the frontal plane. In so doing, the patient mainly innervated the intermediate or acromial fibers of the deltoid (“lateral deltoid”).

### Statistics

For statistical analysis, we used the chi-square test to find significance in categorical and the *t*-test in continuous variables. We tested the MRC measurements of muscle strength using the Sharpino–Wilk test on the normal distribution. Due to non-normal distributed data, the Wilcoxon rank sum test was used to investigate significant differences between the three muscle groups. Since the right and left sides did not show significant differences, they were pooled together to obtain greater power. Furthermore, we analyzed the correlations using Spearman’s correlation test.

A result was considered significant when the *p*-value was < 0.05. To account for multiple testing, the Bonferroni adjustment was applied to each *p*-value. Statistical analyses and the creation of figures were performed using the R software for statistical computing (version 4.2.2, www.r-project.org).

## Results

The average age of the entire cohort (*N* = 71, Table 1) at the time of examination was 63.2 years (standard deviation, SD, 12 years) with an age of onset of 61.5 years (SD 12.4 years). The female/male ratio was 45:55% in the entire cohort, bulbar onset was observed in 20 individuals, whereas spinal onset was observed in 46 cases. Two out of 71 had a positive family history of ALS. BMI at onset was 25.9 (SD 3.45), whereas at the time of examination, it was 24.7 (SD 3.3). The mean Amyotrophic Lateral Sclerosis Functional Rating Scale (ALS/FRS) score at the time of testing was 41.3 (SD 3.83).

**Table 1:** Demographic and clinical characteristics of the cohort

	All patients and muscles	BMRC = 5 in triceps, biceps brachii, and deltoid muscles	BMRC < 5 in at least one muscle (triceps, biceps brachii, and deltoid)
Number of patients	71	38	33
Age of onset (SD)	61.5 (12.4)	65.0 (9.5)	57.8 (14.1)
Age at visit (SD)	63.2 (12.0)	66.52 (8.75)	59.46 (14.1)
Male/female	39/32	17/21	22/11
Onset site			
Bulbar/spinal/thoracic/unknown	20/46/4/1	17/17/3/1	3/29/1/0
Sporadic/familial/unknown	54/2/15	27/0/11	27/2/4
BMI at onset (SD)	25.9 (3.45)	26.0 (3.41)	25.8 (3.57)
BMI at visit (SD)	24.7 (3.3)	24.7 (3.46)	24.7 (3.27)
ALS/FRS (SD)	41.3 (3.83)	41.7 (3.68)	40.8 (4.04)
MRC deltoid muscle			
Right (mean + SD)	4.35 (1.25)	5.0 (0)	3.59 (1.53)
Left (mean + SD)	4.37 (1.33)	5.0 (0)	3.64 (1.68)
MRC biceps brachii			
Right (mean + SD)	4.35 (1.15)	5.0 (0)	3.61 (1.34)
Left (mean + SD)	4.25 (1.24)	5.0 (0)	3.39 (1.4)
MRC triceps brachii			
Right (mean + SD)	4.92 (0.31)	5.0 (0)	4.83 (0.44)
Left (mean + SD)	4.91 (0.41)	5.0 (0)	4.80 (0.59)

*n* = 38 patients, including 17 males and 21 females with a mean age of 66.5 years (SD 8.75), displayed no paresis in the biceps, triceps brachii, or deltoid muscle (Table 1). Seventeen patients reported spinal onset of disease, 17 bulbar, three thoracic onsets, and in one patient the site of onset remained uncertain retrospectively. No patients showed a positive family history. In this group, the patient's BMI decreased on average by 5% following disease onset.

*n* = 33 patients, including individuals displaying at least weakness in one muscle of the six target muscles (BMRC < 5) (Table 1), had a somewhat earlier age of onset (57.8 years, SD 14.1) than the previous group. They were also younger at the time of examination. Both differences were statistically significant (*p* = 0.019 and *p* = 0.012; Chi-square-test).

Not surprisingly, we saw more patients with a spinal onset in the second group (*n* = 29), whereas only three had bulbar onset. In this group, we saw two patients with a positive family history; loss of BMI after disease onset was 4%. The ALS/FRS scores of both groups were comparable (41.7 versus 40.8; *p* = 0.46, *t*-test).

No statistical differences between right and left muscle groups were found for each comparison (biceps: *p* = 0.32; triceps: *p* = 1.00; deltoid: *p* = 0.83). Therefore, we grouped the muscles irrespective of the side examined.

**Fig. 2:**
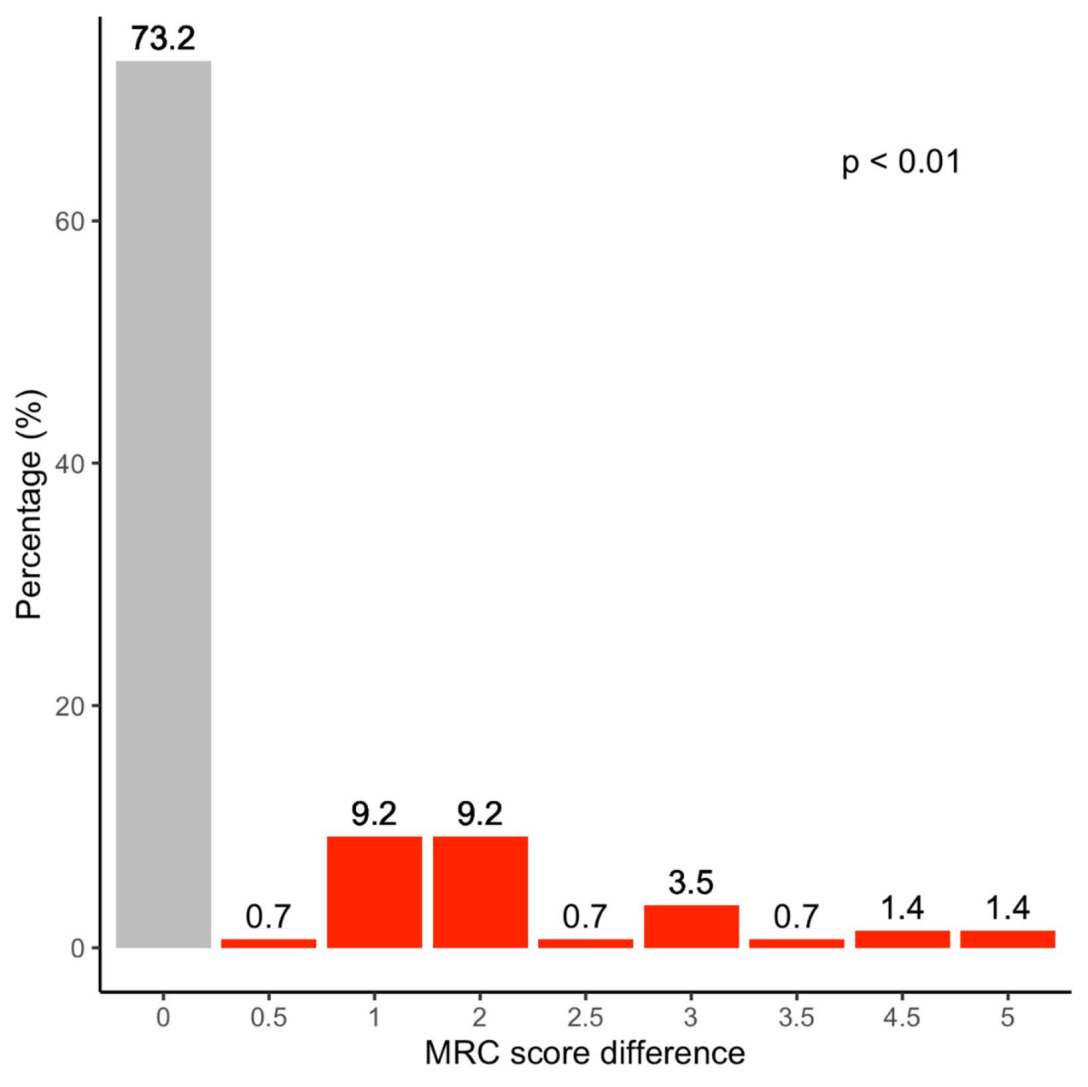
The BMRC Score of the lateral deltoid is compared with elbow extension (triceps brachii) in *N* = 71 patients. In 26.8% of patients, the triceps was stronger than the deltoid, in 73.2% we found no difference, and in no individual was the triceps stronger than the deltoid. The difference attained statistical significance (*p* < 0.01)

**Fig. 3:**
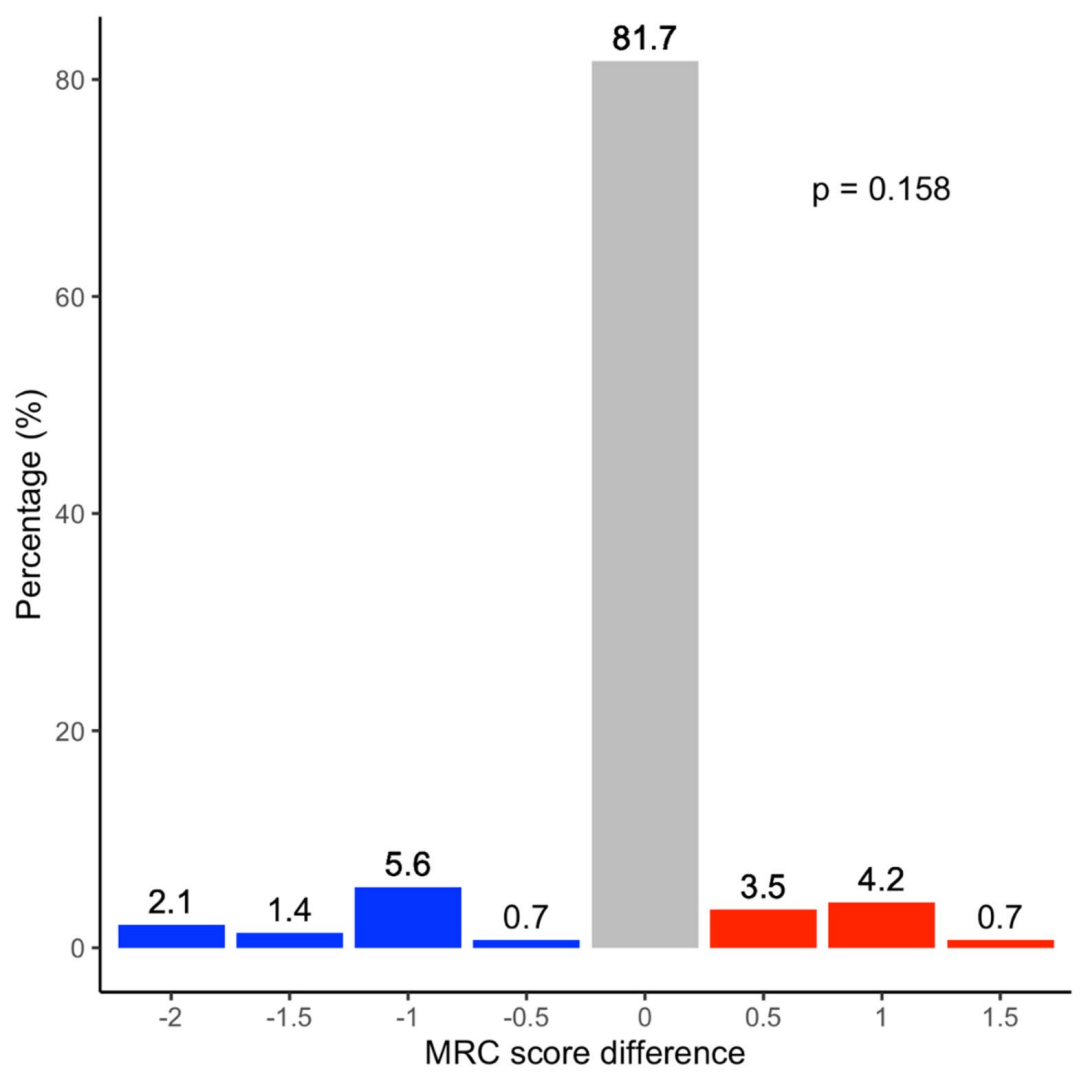
The BMRC Score of the lateral deltoid compared with elbow flexion (biceps brachii) in *N* = 71 patients. In 81.7% of patients, both muscles developed the same strength on the BMRC scale, in 9.8% the biceps was weaker, and in 8.4% the deltoid was weaker. The difference was not statistically significant (*p* = 1.00)

In the first step, we compared the muscle strength (MRC scores) of all muscle groups, including those with an MRC score of 5 in all muscles (no paresis). In all MRC scores, we did not see a normal distribution (Sharpino–Wilk test). Therefore, the Wilcoxon test was used to identify significant differences and Spearman’s test was used to find correlations. The MRC scores of the biceps and the triceps brachii were significantly different (*p* < 0.001). The same was true for the comparison of the deltoid muscle and the triceps brachii (significant difference with a *p* value < 0.001) (Fig. 2). By contrast, the differences between the paresis scores for the deltoid and biceps brachii (Fig. 3) were not different (*p* = 0.16), but also showed a significant positive correlation (*R* = 0.84, *p* < 0.001).

In the second step, we analyzed the scores of those patients who had at least one paretic muscle (MRC < 5; *n* = 33). In summary, we obtained identical results, even in the smaller group, displaying paresis; there was no statistically significant difference between deltoid and biceps (*p* = 0.16) with a positive correlation (*R* = 0.83, *p* < 0.001), but a statistically significant difference between the triceps and biceps (*p* < 0.001) and between the triceps and deltoid muscles (*p* < 0.001).

## Discussion

We previously showed that the patterns of pareses in ALS mirror the pattern of monosynaptic corticospinal connections in nine muscle groups [20]. However, this pattern does not comprise the entire pattern of paresis in ALS. Thus, we examined the deltoid muscle because clinical experience tells us that this muscle is one extremity muscle that is known to become heavily affected early on in the disease process. We compared its strength with the neighboring muscles (biceps and triceps brachii) and found that the loss of deltoid muscle strength resembles the decline of biceps strength rather than the decline of triceps brachii strength.

The MRC grading has to be administered and interpreted with caution because it suffers from several limitations [12, 21]. For this reason, as recommended by MacAvoy & Green [21], we also recorded a percentage of normal (assuming the contralateral side was normal) for each muscle tested.

Our cohort was an early ALS cohort (ALS/FRS > 40). This explains why about one-half of the patients showed normal functioning in the muscles examined. In the first analysis, we included the results of our examination of 426 muscles, the second included 198/426 affected muscles. In each analysis, we could show that the degree of weakness of the biceps and deltoid resembled each other, but the grading of paresis of the biceps or deltoid and triceps was largely different. This result differs from that of Hamada et al. [10] and Sanpei et al. [29], who concluded that, in ALS, the deltoid muscle was weaker than either the biceps or triceps brachii or both; but their findings may be attributable to the fact that the authors grouped (evaluated) the biceps and triceps muscles together.

The strength of the corticospinal connectivity of the deltoid muscle in healthy controls was studied previously by Colebatch et al. [5]. Using electrical and magnetic stimulation techniques of the human cortex, they showed that the deltoid muscle has much greater monosynaptic corticospinal input than the pectoralis major. Clinically, however, the pectoralis major is difficult to study because strength measurements are confounded by the contraction of other muscles. The authors of that electrophysiological study concluded that “the strength of the connections to the deltoid assessed by this method is similar to that of an intrinsic muscle of the hand and significantly larger than that to its antagonist, pectoralis” [5]. A subsequent electrophysiological study by de Noordhout et al. [6] confirmed these findings for the deltoid muscle and also for the pectoralis major and trapezius muscles in humans. Using transcranial magnetic stimulation, in which biceps and deltoid motoneuron pools in controls were more easily and extensively recruited than triceps motoneurons (i.e., biceps > deltoid > triceps brachii) under active conditions, Brouwer & Hopkins-Rosseel [4] concluded that this pattern of activity is consistent with the known pattern of projections of the rapidly conducting primate corticospinal tract [27].

We compared the strength of the deltoid to that of the biceps brachii in ALS. The degree of paresis was similar—much in line with previous electrophysiological studies performed in humans [25, 26]. Our finding is at least complementary to previous findings and their interpretations [1, 7, 14, 20, 31, 32]. It also adds evidence to the hypothesis that muscles monosynaptically supplied by the cortex are more vulnerable to the disease process. In the meantime, it also has been shown that another separate anterior horn cell disease, spinal muscular atrophy, displays a distinct pattern of paresis [9, 30]

Our results are not entirely surprising, given clinical experience and knowledge regarding corticospinal connectivity. The deltoid paresis in ALS is part of a cortical, monosynaptic pattern of paresis. This, in turn, leads to several potential additional implications:

1. How are we to explain the thoracic form of ALS [13] with its two subforms (erector spinae, diaphragm)? The combination of neurophysiological studies of corticospinal connections and neuroradiological quantitative assessment of muscle atrophy [33] might bring this challenge closer to a solution.
2. Similar studies should be performed in bulbar muscles, in particular those of the tongue [23].

## Data Availability

The data that support the findings of this study are obainable from the corresponding author upon reasonable request.
